# Reply to Vilela, R.; Mendoza, L. Current Nomenclature of *Paracoccidioides lobogeorgii*’s Disease Name. Comment on “Grotta et al. Fungal Density in Lobomycosis in French Guiana: A Proposal for a New Clinico-Histological and Therapeutic Classification. *J. Fungi* 2023, *9*, 1005”

**DOI:** 10.3390/jof10010019

**Published:** 2023-12-28

**Authors:** Geoffrey Grotta, Romain Blaizot

**Affiliations:** 1 Dermatology Department, Cayenne Hospital, Cayenne 97306, French Guiana; geoffrey.grotta92@gmail.com; 2 UMR TBIP Tropical Biomes and Immunophysiopathology, University of French Guiana, Cayenne 97306, French Guiana

We have read with interest the comment sent by Raquel Vilela and Leonel Mendoza [1] to our article recently published in the Journal of Fungi, proposing a new classification for Lobomycosis, based on clinical, therapeutic and histopathological grounds [2]. The authors underline the importance of using the new microbiological nomenclature, as the responsible pathogen has recently been renamed *Paracoccidioides lobogeorgii* [3]. The authors also propose using a new disease name, *Paracoccidioidomycosis lobogeorgi* (PCML), or Jorge Lobo’s disease, for lesions caused by *P. lobogeorgii* in humans.

Following the important work by the same team (Vilela et al.) in 2023 in reviewing the taxonomy of *Lacazia loboi* and proposing its renaming as *P. lobegeorgii*, we chose to use this pathogen name so as to follow the proper nomenclatural rules. On the other hand, we saw no proposal in this article concerning the disease’s name [3]. The name currently registered by the International Classification of Diseases (ICD) is still Lobomycosis (B48.0) [4]. Since the publication of Vilela et al.’s article, it has been used in several papers including a first report in Panama [5].

Indeed, pathogens and disease names do not follow the same rules. Guidelines have been proposed by the World Health Organization (WHO) in naming diseases properly, which include avoiding personal, geographic or animal names [6]. Therefore, it seems relevant to stop calling this disease “Lobomycosis”, as “lobo” means “wolf” in Portuguese and could lead to misinterpretations in Brazil, the country with the largest number of reported cases. However, using “George Lobo” in the name of the disease does not seem to comply with these guidelines.

In our opinion, disease names should reflect the nosological and clinical aspects. Lobomycosis/PCML/Jorge Lobo’s disease presents a very different clinical pattern from the disease caused by cultivable strains of *Paracoccidioides* and which is known as Paracoccidioidomycosis (PCM) [7]. Renaming lobomycosis as PCML, in our opinion, does not highlight the clinical differences between cultivable and uncultivable *Paracoccidioides* strains and seems to consider Lobomycosis/PCML/Jorge Lobo’s disease as a mere subtype of PCM. Lobomycosis involves only the skin and more rarely the lymph nodes, while PCM is mostly a systemic and disseminated disease involving many organs [2,7]. The cutaneous lesions caused by PCM are various and nonspecific, while the keloid-like lesions of Lobomycosis are typical (though other, less specific cutaneous lesions can also be found in Lobomycosis). The treatment of Lobomycosis and PCM are also very different [2,8]. Though from a microbiological point of view, these two entities are caused by pathogens belonging to the same genus, from a clinician’s point of view, they represent two different clinical diseases and their names should reflect it. 

Other specific aspects of Lobomycosis include its uncultivable nature and its location in South America. However, it is not impossible that a specific growth medium would be designed in the future to allow the culture of these strains, which probably require phagocytosis to develop. Concerning the geographical locations, names of areas and countries should be avoided in naming diseases in order to avoid any stigmatization. Besides, the endemic area of Lobomycosis could be wider than expected, as suggested by a recent report in Panama. 

We suggest that the strictly cutaneous nature of Lobomycosis should be highlighted in its new name, as this is the most significant feature of this disease when compared to PCM. We suggest considering *Strictly Cutaneous Paracoccidioidomycosis* (SCP) or *Paracoccidiodermatosis*.

In any case, the process of renaming this disease should be undertaken by the different experts involved in its management in South America. It is important that microbiologists, clinicians and public health experts working on Lobomycosis/PCML/Jorge Lobo’s disease get involved so as to ensure that all potential authors use the same names. This process should end up with a provisional name before officially changing the WHO/ICD nomenclature.
